# Genome-wide identification of the TIFY family reveals JAZ subfamily function in response to hormone treatment in *Betula platyphylla*

**DOI:** 10.1186/s12870-023-04138-6

**Published:** 2023-03-15

**Authors:** Guanbin Lv, Rui Han, Jingjing Shi, Kun Chen, Guifeng Liu, Qibin Yu, Chuanping Yang, Jing Jiang

**Affiliations:** 1 State Key Laboratory of Tree Genetics and Breeding, Northeast Forestry University, Harbin, 150036 China; 2 College of Forestry and Grassland Science, Jilin Agricultural University, Jilin, China; 3 University of Florida, Lake Alfred, FL USA

**Keywords:** *Betula platyphylla*, *TIFY*, JAZ, Phylogenetic analysis, Expression profile, Protein interaction

## Abstract

**Background:**

The TIFY family is a plant-specific gene family and plays an important role in plant growth and development. But few reports have been reported on the phylogenetic analysis and gene expression profiling of TIFY family genes in birch (*Betula platyphylla*).

**Results:**

In this study, we characterized TIFY family and identified 12 TIFY genes and using phylogeny and chromosome mapping analysis in birch. TIFY family members were divided into JAZ, ZML, PPD and TIFY subfamilies. Phylogenetic analysis revealed that 12 TIFY genes were clustered into six evolutionary branches. The chromosome distribution showed that 12 TIFY genes were unevenly distributed on 5 chromosomes. Some TIFY family members were derived from gene duplication in birch. We found that six JAZ genes from JAZ subfamily played essential roles in response to Methyl jasmonate (MeJA), the JAZ genes were correlated with COI1 under MeJA. Co-expression and GO enrichment analysis further revealed that JAZ genes were related to hormone. JAZ proteins involved in the ABA and SA pathways. Subcellular localization experiments confirmed that the JAZ proteins were localized in the nucleus. Yeast two-hybrid assay showed that the JAZ proteins may form homologous or heterodimers to regulate hormones.

**Conclusion:**

Our results provided novel insights into biological function of TIFY family and JAZ subfamily in birch. It provides the theoretical reference for in-depth analysis of plant hormone and molecular breeding design for resistance.

**Supplementary Information:**

The online version contains supplementary material available at 10.1186/s12870-023-04138-6.

## Background

The TIFY gene family is a unique family in plants and its protein contains a special highly conserved domain TIFY which consists of about 36 amino acids [1]. TIFY gene family was named for the conserved sequence TIF[F/Y]XG [2]. The TIFY gene was identified in *Arabidopsis*(*Arabidopsis thaliana*) [3], rice (*Oryza sativa*) [4] and wheat (*Triticum aestivum* L) [5]. TIFY protein family was divided into four subfamilies based on conserved sequence domains, including Zinc-finger inflorescence meristem (ZIM/ZML), TIFY-domain, Jasmonate ZIM-domain (JAZ), and PEAPOD (PPD) [6]. PPD proteins contain TIFY, JAS and PPD domain. JAZ proteins contain TIFY and JAS domain. ZML proteins contain TIFY, CCT and C2C2-GATA zinc finger domain, but TIFY subfamilies only contain TIFY [7]. TIFY family genes were involved in hormone response and plant development. In rice, overexpression of *TIFY11a* (or *JAZ9*) can alleviate growth inhibition through a stress-inducible promoter under the treatment of salt and dehydration stresses [4]. In *Arabidopsis*, the TIFY proteins can be induced by high salt, ozone and mechanical damage. Mutant seeds with knocking out *AtTIFY10a* and *AtTIFY10b* genes had relatively low germination rate under alkaline stress. This demonstrated that *AtTIFY10s* played positive roles in response to alkaline stress [8]. The absence of the PPD locus increased the size of the leaf, resulting in a dome-shaped rather than flat-shaped leaf in *Arabidopsis* [9].

In regulating the growth and response to biotic and abiotic stresses, phytohormones play a major role in plants. Previous studies indicated that jasmonic acid (JA), abscisic acid (ABA) and salicylic acid (SA) were the main hormones response to the stress [10, 11]. JA and Jasmonates (JAs) are lipid derivatives produced by lipoxygenase (LOX)-mediated oxidation of unsaturated fatty acids [12]. Under normal circumstances, Jasmonic acid in plants maintained at a low level. However, under stress, JA signal molecules were produced in large quantities, and JA-Ile complexes were formed under the activity of JAR1 enzyme. Isoleucine conjugate (JA-Ile) promoted the binding of SCF^COI1^ complex and JAZ proteins to hydrolyze 26S protease and JAZ proteins. MYC2 and other transcription factors were released to activate JA signal transduction pathway. Therefore, JAZ protein plays an important role in the production of JA signal molecules. *AtJAZ3* and *AtJAZ9* were involved in JA-mediated synthesis [13]. JAZ subfamily proteins were more associated with the response to abiotic and biotic stresses than other subfamilies [14]. JAZ proteins function as repressors, involve in a variety of signal pathways and negatively regulate JA. He et al. cloned a key inhibitor *GhJAZ2* and overexpression of *GhJAZ2* weakened cotton's sensitivity to JA and resistance to the fungus Verticillium (*Verticillium dahlia*) and cotton bollworm [15]. JAZ proteins don’t have an identifiable DNA binding domain [16]. They can form homologous or heterodimers with themselves or other JAZ proteins or binds to other transcription inhibitors [3]. JAZ proteins are the direct target of SCF^COI1^ complex which is constituted by COI protein, SKP1, cullin1 and Rbx1. COI1 is the receptor of JA-Ile and is an important part of SCF (Skp-Cullin-F-box) complex [17, 18].The first COI1 protein was cloned from *Arabidopsis* [19]. It encodes an F-box protein that regulates plant response to stress and plant growth [20]. Therefore, it is an important to understand the relationship between JAZ proteins and COI1 protein in JA signaling pathway.birch belongs to deciduous broad-leaved tree species. It grows faster and has strong cold tolerance and certain medicinal value. Therefore, understanding the mechanism of biotic and abiotic stress in birch is of great significance. Previous studies have shown that members of the JAZ subfamily and the COI protein are all related to JA [21, 22]. In birch, our previous study found that reduced *BpCOI1* expression leads to reduced ability to respond to exogenous Methyl jasmonate (MeJA) signals [23]. Overexpression *COI1* plant was represented by OE, Inhibition expression *COI1* plant was represented by IE and wild-type plant is represented by WT. In this study, we characterized TIFY family genes using phylogeny and chromosome mapping analysis, explored the differential expression patterns of TIFY genes in response to MeJA treatment and the relationship between members of the JAZ subfamily and *BpCOI1*. We also explored the JAZs proteins in response to differential hormone treatments and the relationship of JAZ proteins to explain its regulation of hormones. Our results provided novel insights into biological function of brich TIFY family and JAZ subfamily.

## Results

### Identification of the TIFY gene family and phylogenetic analysis

**Fig. 1:**
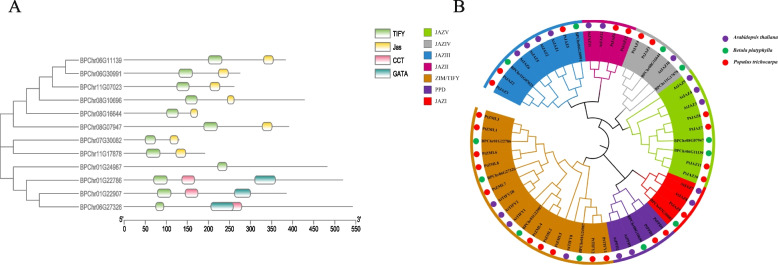
**A** Visualization of conserved domains of TIFY protein family in birch; **B** Dendrogram of birch, poplar and Arabidopsis TIFY members. The dendrogram was drew by MEGA7 with the neighbor joining method. Different groups are marked with different colors

We identified 12 birch TIFY genes by HMMER analysis (E-value < 1 × 10^–3^), the genomic DNA, CDS and protein sequences of all BpTIFY members as data S1, S2, and S3. The domain sequences of birch TIFY family are showed in Table S1, and the domain locations are displayed in Fig. 1A. The results showed that all proteins contained TIFY domain. BPChr01G24987 only contains a TIFY domain but no other motif was assigned as a member of the TIFY subfamily. The proteins containing the TIFY domain and the CCT and/or GATA motif were classified as the ZML subfamily [24, 25]. The three proteins contain TIFY domain, CCT domain and GATA domain, which three proteins were assigned as member of the ZML subfamily. Although there were eight proteins containing TIFY domain and JAS domain, one of them with a Jas motif lacked the conserved PY motif at its C-termini, which is characteristic of a partial Jas domain in PPD proteins. To identify the conserved domain of PPD, we selected two members from the PPD subfamily of Arabidopsis, and performed multiple sequence alignments. The results indicated that BPChr08G10696 contains a conserved PPD domain of 156 amino acids that harbors a highly conserved sequence at the N-terminus (Figure S1) [4, 26, 27]. And other seven proteins were assigned as member of the JAZ subfamily. As shown in Table S2, 1 gene belongs to TIFY subfamily, 1 gene belongs to PPD subfamily, 3 genes belong to ZML subfamily and 7 genes belong to JAZ subfamily.

The constructed phylogenetic tree using the TIFY protein sequence was shown in the Fig. 1B. All TIFY proteins were clustered into seven branches. ZML and TIFY proteins were in one branch; PPD proteins were in another unique branch. The protein containing the JAS domain was clustered into five branches (I to V); This result was consistent with previous studies on *Salvia miltiorrhiza*, *Brassica napus* and *Populus pilosa* protein [28–30]. In addition, we also made Maximum Likelihood tree (Figure S2) and Bayesian phylogenies (Figure S3), and found that the grouping is similar to NJ evolutionary trees. The result indicated that these proteins had a broader phylogenetic relationship, and these species diverged from each other and experienced significant mutations in the early stages of the evolutionary process of terrestrial plants. In each branch, we observed that poplar and birch were closer in smaller branches than *Arabidopsis*. These results indicate that the genetic relationship between birch and poplar was closer than *Arabidopsis*.

### Gene sequence analysis and prediction of cis-elements in BpTIFYs promoter region

**Fig. 2:**
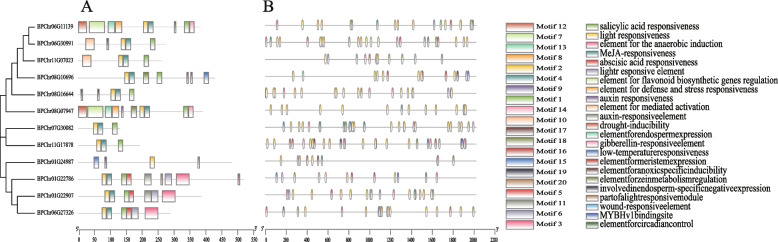
Gene motif and promoter element compositions of the birch TIFY members. **A** Conserved motifs in the TIFY members. The motifs were identified by the MEME Suite. The motif logos were drawn by use of the Tbtools. The colorful boxes represent different conserved motifs; **B** Promoter elements in the TIFY members. The elements were identified by the PlantCARE. The elements logos were drawn by use of the Tbtools. The colorful boxes represent different elements

Exploring the conservative motifs in 12 TIFY proteins of birch using MEME software [31]. As shown in Fig. 2A and Table S1, 20 conserved motifs were found by MEME. In total, 20 conserved protein motifs were annotated: motif 2 as CCT, motifs 7,8 as TIFY domain, motif 12 as Jas domain, motif 13 as GATA domain. The rest had no annotation. The prediction showed that BPChr01G24987 did not have Motif 1, Motif 4, and all the others had Motif 2 and Motif 4, which may be the difference between the TIFY subfamily and other subfamilies. Some proteins have similar motifs (BPChr01G22907, BPChr01G22786 and BPChr06G27326) (BPChr06G11139, BPChr08G07947) (BPChr06G30991, BPChr11G07023), which indicated that these proteins had similar functions.

The cis-acting elements are the key regions of intergenic regulation. In plants, Proteins can induce, enhance gene transcription by binding to specific cis-acting elements [32] The website of PlantCARE (http://bioinformatics.psb.ugent.be/webtools/plantcare/html/) was used to analyzed the *BpTIFY* promoter to obtain cis-acting elements. As shown in Fig. 2B, five hormone-related and five stress-related components were identified in 2 kb upstream regions of *BpTIFY* genes. Hormone related elements include AuxRR core/ Auxre/ TGA element (response to auxin), GARE-motif / P-box (response to gibberellin), TCA element (response to salicylic acid), TGACG motif/ CGTCA motif (response to JA) and ABRE (response to ABA). We found that 8 TIFY promoters contained ABRE, which is an important regulatory element in response to ABA. Seven TIFY promoters contained TGACG motif or CGTCA motif, which was an important regulatory element in response to MeJA. Six TIFY promoters contained TCA element, which was an important regulatory element in response to SA. BpTIFY family probably participates in plant hormone pathway. Some stress-related elements include MBS (response to drought-inducibility), LTR (response to low-temperature), TC-rich repeats (defense and stress responsiveness), WUN-motif (wound-responsive element). Result showed that BpTIFY family probably was involved in the stress response. According to these results, BpTIFY family probably affect that Plants exhibit responses against biotic and abiotic stresses by regulating. hormone.

### Chromosome distribution and gene duplication

**Fig. 3:**
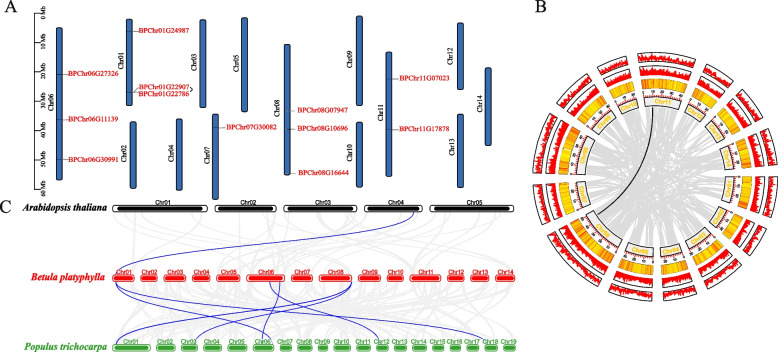
Chromosome distribution and gene duplication. **A** Chromosome distribution of birch TIFY genes. Chr01–14 represent chromosome numbers 01–14. **B** Collinearity analysis of the TIFY gene family in birch. Chromosomes 01–14 are represented by rectangles. The lines, heatmaps, and histograms along the rectangles represent gene density on the chromosomes. The gray lines indicate synteny blocks in the birch genome, while the lines of other colors between chromosomes delineate segmental duplicated gene pairs; **C** Synteny analysis of the TIFY genes between birch and two other plant species. The gray lines indicate gene blocks in birch that are orthologous to the other genomes. The blue lines delineate the syntenic TIFY gene pairs

The chromosome distribution of birch TIFY family was shown in Fig. 3A and Supplementary Table 4. Birch 12 TIFY genes were unevenly distributed on 5 chromosomes of total 14 chromosomes. In this study, TIFY family members were named sequentially according to their chromosomal location and subfamily (Table S3). Gene duplication events play a crucial role in the formation of gene families. Segmental duplication and tandem duplication are main driving forces of gene duplication [33]. Using TBtools and MCScanX examined the tandem duplication events to understand the expansion of the TIFY family genes in birch. Two genes on chromosome 1 constituted a tandem replication and the events occur in the same region (Fig. 3A and Table S4).

In addition, we also used BLASTP, MCScanX and TBtools to identify fragment duplication events. As were shown in Fig. 3B and Table S4, among 12 genes distributed on 5 chromosomes, a total of one gene pair was observed to have fragment duplication event. These two genes belong to the BpJAZ subfamily, so results suggest that fragment duplication may play a critical role in the gene duplication events of the BpJAZ subfamily in birch.

We constructed a collinearity map to observe TIFY gene family by comparing the sequence similarity between birch, two species of *Arabidopsis* and poplar (Fig. 3C and Table S5). A total of four birch genes had a collinearity relationship with one *Arabidopsis* gene and six poplar genes. Six orthologs between birch and poplar were identified, far more than that between birch and *Arabidopsis*. Probably because both birch and poplar are woody plants. It is worth noting that we found that the four birch TIFY genes have collinear relationships with six genes in poplar. We believed that in different species, these genes might share same important functions.

### *TIFY* family gene expression under MeJA treatments

**Fig. 4:**
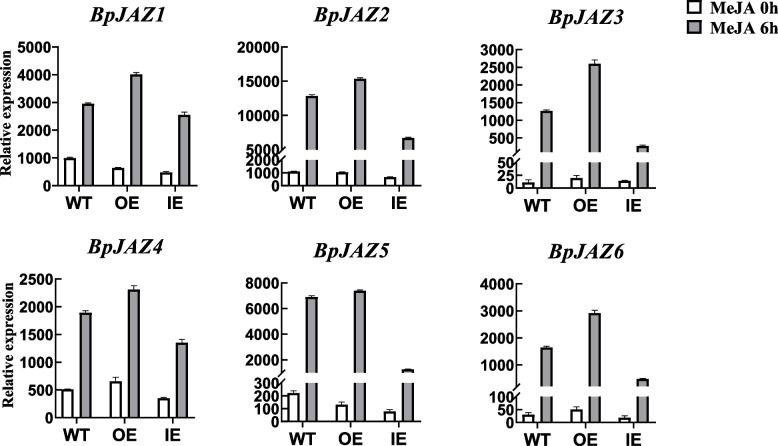
Gene expression levels based on RNA-Seq

Same six DEGs were identified both in WT and IE lines whereas seven DEGs were identified in the OE lines. All of them belonged to JAZ subfamily and they were up-regulated. After MeJA treatments, the expression level of these genes in IE lines was significantly decreased than OE lines and WT lines. It suggested that the expression level may be related to the expression of COI gene in plants. The results of RNA-Seq as shown in Fig. 4.

### Co-expression networks and gene ontology analyses

**Fig. 5:**
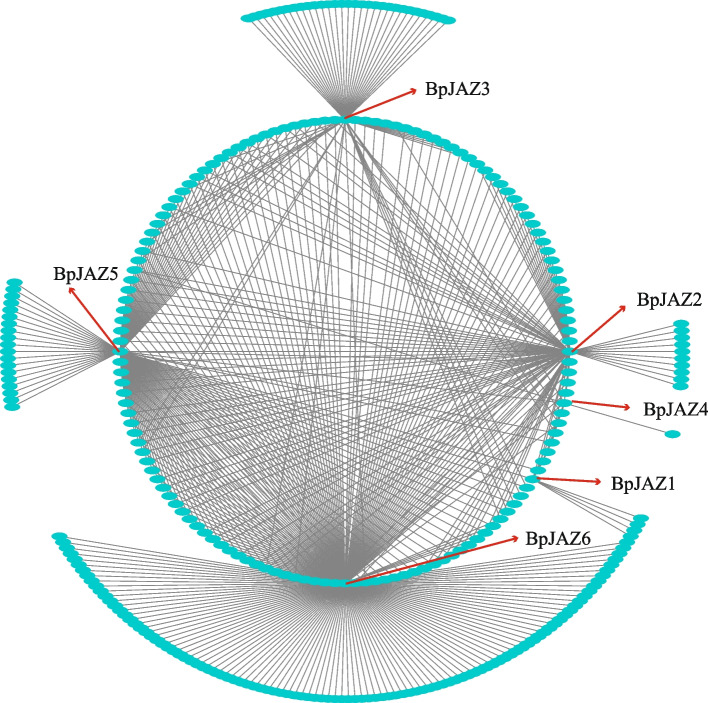
TF-centered co-expression network of six tissue differentially expressed genes. Dots represent genes, and lines indicate that they have co-expression relationship

Co-expression analysis can help us find gene expression patterns with similar characteristics. These genes may affect the same physiological process or hormone pathway, and they are functionally related. We used the WT, IE transcriptome data after MeJA stress constructed a co-expression network with 6 *BpJAZ* genes, we obtained a co-expression networks (Fig. 5). We selected genes with correlation coefficient values 1 and found that the co-expression networks of the six *BpJAZ* genes were similar in size. The results showed that there may be a redundant relationship between the physiological functions of these genes in birch.

**Fig. 6:**
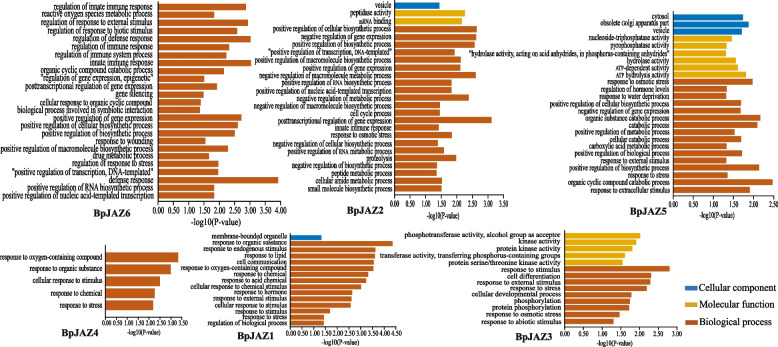
Go enrichment analysis of six co-expressed gene sets

To explore the biological processes of these genes, we performed gene set enrichment analysis on 6 groups of co-expressed genes (Fig. 6). These six *BpJAZ* genes contain some GO terms, such as response to hormone, stimulus, stress and wounding. This indicated that six *BpJAZ* genes play an important role in stress. These genes also contain some GO terms related to plant growth and development, such as biosynthetic process, secondary metabolite biosynthetic process, membrane-bounded organelle. Therefore, it was speculated that the *BpJAZ* genes play an important role in birch growth process in response to external stress.

### *BpJAZ* genes expression under hormone treatments

**Fig. 7:**
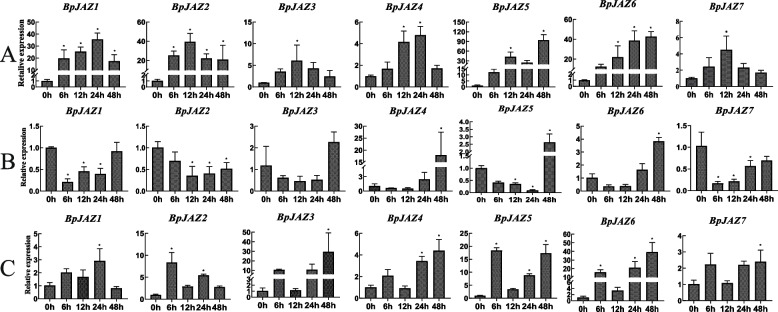
Gene expression levels of JAZ genes under different hormone treatment by qRT-PCR. Statistical analysis was performed using one-way ANOVA, significant differences (*P* < 0.05) are indicated by *. Three biological replicates were used. **A** Gene expression levels after MeJA treatment. Error bars are standard deviations from the biologic replicates; **B** Gene expression levels after ABA treatment. Error bars are standard deviations from the biologic replicates; **C** Gene expression levels after SA treatment. Error bars are standard deviations from the biologic replicates

To better explore the *BpJAZ* gene functions, seven *BpJAZ* genes expressions of 6-week-old plants were measured under treatments of MeJA, SA and ABA for 0 h, 6 h, 12 h, 24 h, and 48 h. Under MeJA treatment, the seven genes were all positively correlated (Fig. 7A). The gene expression levels of the seven *BpJAZ* genes showed that there were different trends at different time points under SA stress. However, most genes were negatively correlated between 0 h-24 h, and these increased gene expressions during 24 h-48 indicated positive regulation (Fig. 7B). Under ABA treatment, the seven *BpJAZ* genes were all positively correlated. Their expression levels increased after in 6 h treatment, then decreased rapidly at 6 h-12 h and increased again (Fig. 7C). Combining with the results of MeJA, SA and ABA treatments, we found that the JAZ subfamily genes were not only related to the JA pathway, but also involved in the regulation of the ABA and SA pathways.

### Subcellular localization and yeast two-hybrid

**Fig. 8:**
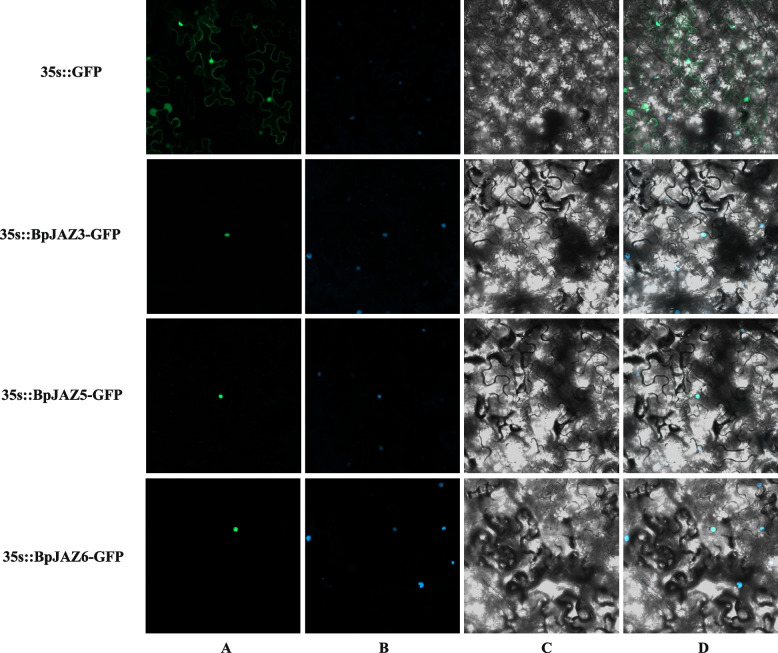
Subcellular localization analysis of BpJAZ. We transferred the constructed fusion vector (35S:BpJAZs-GFP) and control vector (35S:GFP) into tobacco by injection. **A** GFP fluorescence; **B** DAPI staining **C** bright fields; **D **combined images of GFP

To verify the interaction between JAZ proteins, we did the yeast two-hybrid. First, we conducted subcellular localization experiments on these 3 BpJAZ proteins. The subcellular localization of the JAZ family proteins were transiently expressed in the tobacco leaves (Fig. 8). The fluorescent signal of 35S:GFP protein can be observed in the whole cell. The fluorescence signal of BpJAZ3, BpJAZ5, and BpJAZ6 were observed in the nucleus. Therefore, BpJAZ3, BpJAZ5 and BpJAZ6 were nuclear localization proteins.

We used the constructed vector to perform yeast two-hybrid. The positive control, negative control and 9 recombinant vectors grew on SD/-Trp/-Leu solid medium which indicated that all plasmid combinations were successfully transferred into yeast cells. All the combinations can grow on the SD/-Trp/-Leu/-His/-Ade/X-α-Gal/AbA (200 ng/mL) solid medium except pGBKT7-BpJAZ3/pGADT7-BpJAZ3 (Figure S4). The results confirmed that BpJAZ3, BpJAZ5, BpJAZ6 can form homodimers, and BpJAZ3, BpJAZ5, BpJAZ6 can form heterodimers together. This indicates that BpJAZs proteins function in the form of homodimers or heterodimers.

## Discussion

### Characterization of the birch TIFY gene family

The TIFY family in plants has been identified in plants such as *Arabidopsis*, rice, wheat [3–5]. In this study, 12 TIFY genes were identified in birch. 1 gene belongs to TIFY subfamily, 1 gene belongs to PPD subfamily, 3 genes belong to ZML subfamily and 7 genes belong to JAZ subfamily. Phylogenetic tree of the TIFY proteins of *Arabidopsis*, poplar and birch revealed that TIFY family genes in birch are clustered into seven groups, which are consistent with those in *Arabidopsis* and poplar. According to the function of TIFY protein in *Arabidopsis* and Poplar, we can speculate TIFY protein function of birch. This phenomenon deserves further studies.

TIFY family genes had their diverse structural domains and were involved in hormone response and plant development [14]. In this study, several conserved domains, such as CCT, TIFY, Jas and GATA were found at the TIFY proteins. The motif structure in each TIFY subfamily had mostly conservative domains, which refers to the conservative and specific functions of proteins in these subfamily. Our result is consistent with previous studies [14].

Promoters regulate gene expression by controlling the starting frequency and the efficiency [34]. Sequence analysis of promoters clarified the role of gene function in adapting to adversity environments [35]. In our study, we found that TIFY genes contained many *cis*-acting elements, such as ABRE, TCA element, GARE-motif, TGACG motif, MBS, TC-rich repeats and WUN-motif, which was related to various hormones and stress. Among these, ABRE was the most abundant hormone response element, which was the major cis-element for ABA-responsive gene expression can regulate stress-response and seed development [36]. TGACG motif was the major cis-element in TIFY genes, which for JA-responsive gene expression [37]. MBS was an important binding site of MYB transcription factors and regulates downstream genes through mutual binding under drought stress [38]. The WUN-motif has been reported to induced in response to wounding [39]. previous studies indicated that BpTIFY family probably affects the defense of plants against biotic and abiotic stresses by regulating hormone [40]. In this study, we found that this may be related to TIFY family genes containing these cis-acting elements.

Expanding family members through repeated events is the basis for maintaining the stability of the extended family in the evolutionary process [41], such as F-box [33], bZIP [42]. In this study, the TIFY genes in birch were distributed on five chromosomes. One gene pair had segmental duplication event and another gene pair had fragment duplication event. These results showed that the segmental duplication may play a critical role in the gene duplication events of the BpZML subfamily. Fragment duplication may play a critical role in the gene duplication events of the BpJAZ subfamily in birch which may reveal the direction of replication gene expansion. Interestingly, the TIFY gene replication event of birch had strict replication criteria, which only occurred in its subfamily. We also explored the collinearity of the TIFY genes among birch, *Arabidopsis* and poplar. A total of four birch genes has a collinearity relationship with one *Arabidopsis* gene and six poplar genes. There were six orthologs between birch and poplar, far more than *Arabidopsis,* which may indicate that poplar and birch are more closely related. Our results provide insights into the evolutionary relationship of TIFY family genes in birch and other species.

### JAZ subfamily in response to hormone treatment

TIFY proteins play an important role in abiotic stress response, especially the JAZ protein which is related to JA signal transduction and it related to plant disease resistance and defense. He Y et al. (2020) found that rice overexpressing *OsJAZ4* plants negatively regulates JA signal and antiviral defense [43]. Methyl jasmonate (MeJA), a natural phytohormone, played a critical role not only in plant growth but also in plant defense response to biotic and abiotic stresses [44]. Therefore, it is possible to study JAZ family through exogenous application of MeJA to induce plant disease resistance and defense response. Under MeJA stress, we found that only the JAZ subfamily were regulated by the stress in the TIFY family. Previous studies showed that members of the JAZ subfamily of the TIFY family and the COI protein are all related to JA [21, 22]. The function of COI1 is indispensable in JA signal pathway. The mutation of COI1 gene leads to the lack of JA in plants. Jia et al. (2016) studied the genes related to grape JA synthesis and signal receptors and found that the expression of JA signal receptor gene *VvCOI1* increased after treatment with exogenous MeJA in grape [45]. The transgenic strawberry enhanced the resistance to *Botrytis cinerea* [45]. Under MeJA stress, the expression level of *BpCOI1* in the IE lines was significantly lower than that of WT and OE lines. This may be related to reduce the expression of COI protein in plants. Lee et al. found a specific interaction between OsCOI and OsJAZs in rice [21].

To further explore the function of BpJAZ subfamily, we analyzed the co-expression network of these proteins, and then enriched the co-expression genes of JAZ proteins. We found that the JAZ genes are related to plant hormones, growth and development. The expression levels of JAZ genes were all positively correlated under MeJA treatments in 0 h, 6 h, 12 h, 24 h, and 48 h. This result is similar to previous studies [24, 28, 29].

In this study, the JAZ genes were not only related to the MeJA, but also involved in the regulation of the ABA and SA. The JAZ genes were up-regulated after MeJA treatment, while the expression of JAZ genes were down regulated after SA treatment. We speculated that JA and SA have a certain antagonistic effect in birch. Some studies also found that there was an interaction between SA and JA biosynthesis pathways [46]. In this study, we also found that JAZ genes were up-regulated after MeJA and ABA treatment. Previous studies showed that JA and ABA have the same gene expression regulation system [47]. In plants, JAZ protein does not regulate JA alone, but regulates other plant hormone signal pathways to form complex signal networks together. The defense against abiotic stresses, including drought, high salt, cold and other environmental conditions, will cause the increase of ABA levels in plants [36, 48, 49]. ABA is a critical phytohormone involved in multifaceted processes in plant metabolism and growth under both stressed and nonstressed conditions [50]. Plant hormones play an important role in mediating plant defense against microbial pathogens, including SA, JA, ET [51]. Therefore, it is explained that JAZ genes are involved in plant growth, development and response to biotic and abiotic stresses.

Through the analysis of gene co-expression networks and gene ontology, it was found that there was a redundant relationship between the co-expression genes of JAZ. It can be seen that the JAZ genes were correlated to various plant hormones. The ZIM domain is a protein–protein interaction domain that mediates homologous and heterologous interactions between JAZ proteins [52]. Therefore, the JAZ protein may interact with itself or other transcription factors. To verify the interaction between JAZ proteins, we chose BpJAZ3, BpJAZ5, and BpJAZ6 with highest expression levels under MeJA stress. First, we verified its subcellular localization. In subcellular localization experiments on the 3 proteins of the JAZ subfamily, we found that they were localized in the nucleus. The result is consistent with previous studies [26, 53, 54]. The interaction relationship was verified and had proved that BpJAZ3, BpJAZ5 and BpJAZ6 can form homodimers, while BpJAZ3, BpJAZ5, and BpJAZ6 can form heterodimers with each other. Previous hypothesis suggested that the homodimers of JAZs may contribute to the stability of proteins, and the heterodimers also facilitate the simultaneous interaction of multiple JAZs with MYC proteins [24, 55]. In this study, we found that the 6 genes of the JAZ subfamily play an important role in regulating various plant hormone pathways by forming homodimers and heterodimers and other genes.

## Methods

### Member of TIFY family and construction of phylogenetic tree

The amino acid sequences of birch proteins were downloaded from the phytozome website (https://phytozome.jgi.doe.gov/) [56] and that of TIFY (PF06200) were from the Pfam database (https://pfam.xfam.org) [25, 57]. We used hmmsearch (http://www.hmmer.org/) with TIFY to search the birch amino acid sequences with a threshold of E value < 1 × 10^–3^. We used SMART databases (http://smart.embl.de/) and the NCBI web CD-search tool (https://www.ncbi.nlm.nih.gov/Structure/bwrpsb/bwrpsb.cgi) to test [58]. To identify the conserved domain of PPD, Multiple sequence alignment of *Arabidopsis* [59] and birch PPD proteins was performed using ClustalX 1.83. Phylogenetic tree was constructed based on amino acid sequences of birch, *Arabidopsis* and poplar. Neighbor-Joining tree by was constructed using neighbor joining method of MEGAX with 1000 repeated bootstrap test [30]. Using MEGA X, we constructed a phylogenetic tree with the Maximum Likelihood method, 1000 repetitions of bootstrap tests, and JTT matrix based model [60, 61]. The nucleotide sequences were aligned by usie of BioEdit software [62]. MrBayes 3.2.7 was used to construct Bayesian phylogenies [63]. The BEASTmodels analysis was performed using MEGAX. The stationary distribution of the MCMC chains and the convergence of runs were monitored using Tracer (v.1.6) to determine the appropriate MCMC chain length such that the effective sample size of every parameter was larger than 200 as recommended. Tree pictures were generated using TreeViewX. We also used TBtools software to visualize the evolutionary tree of conserved motifs and their domains.

### Identification and sequence analysis on the TIFY members

The genome sequences to analyze the birch TIFY family, the phytozome website (https://phytozome.jgi.doe.gov/) to use downloaded the sequences.

 [56]. The conserved motifs in the TIFY proteins were identified by the MEME Suite [31]. Birch promoter sequences were downloaded from online website (https://pubmed.ncbi.nlm.nih.gov/33574224/) [56]. The PlantCARE was used it to analyze the birch TIFY family promoters (http://bioinformatics.psb.ugent.be/webtools/plantcare/html/) [64]. TBtools was used to visualize the promoter elements [65].

### Chromosome distribution and gene duplication

The birch genome data downloaded from the Phytozome database (https://phytozome.jgi.doe.gov/). TIFY family location on chromosome was visualized using the annotation information of the birch genome by TBtools [65]. We analyzed the tandem duplication events of the TIFY gene family and investigated segmental duplication events and the collinearity relationship for gene pairs from different species by using MCScanX and BLASTP methods in TBtools [41].

### Plant materials and gene expression analysis

In birch, we analyzed the RNA-Seq data to characterize the response of TIFY family to MeJA. The transformation constructs of overexpression vector and RNAi inhibitory expression vector were introduced into EHA105 strain and used to infect birch zygotic embryos [66]. The infected zygotic embryos were co-cultivated in the dark for 2–3 days and were then transferred to selection medium until the transgenic lines were obtained [67]. Group1 and Group2 were selected as test materials. Group1: the wild type (WT), overexpression transgenic *BpCOI1* lines (OE) and RNAi-inhibited expression transgenic *BpCOI1* (IE) lines were treated with 100 μmol MeJA for 6 h. Group2: the WT, OE, IE lines irrigated with water were used as control. The expression level of genes were calculated by RNA-Seq method [68]. The differentially expressed genes (DEGs) of treated plants were identified using DESeq software and the thresholds were fold change ≥ 2 and padj (p-value adjusted for multiple testing) <  = 0.05 [69].

### Gene co-expression networks and gene ontology analyses

We constructed the co-expression network of JAZ genes using Spearman method [70]. The genes were used to construct co-expression networks with correlation coefficient greater than 0.9. Correlation coefficient was calculated using Pearson algorithm. Results were visualized by Cytoscape [71]. The TBtools was used to study the Gene set enrichment analysis (P-value < 0.05).

### BpJAZ genes expression under hormone treatments

We selected one-month-old wild-type seedling with similar growth status, which were divided into four equal groups with three biological replicates. Groupn1, Group 2 and Group 3 were treated with 100 μmol/L MeJA, 100 μmol/L SA and 100 μmol/L ABA for 0 h, 6 h, 12 h, 24 h, 48 h, respectively. Three plants at each time point were used as biological replicates. Group 4 was used as a control. Total RNA was extracted and reversed transcripted into cDNA. qRT-PCR was performed with cDNA as a template to calculate the relative gene expression of JAZs family. qRT-PCR experiment was performed by 7500 real-time fluorescent quantitative PCR instrument (ABI) with SYBR Green Real time PCR Master Mix (Japan TOYOBO) and procedures were carried out according to the product protocols (Japan TOYOBO). Primer sequences for qRT-PCR were listed in Table S6. We used the 2^−ΔΔCt^ method to analyze qRT-PCR results [72].

### Subcellular localization and yeast two-hybrid experiment

Primers were designed based on transcript sequences of the *JAZ* family. Primer sequences are shown in Table S6. The pBI121-BpJAZ-GFP fusion expression vector was constructed and PBI121-BpJAZ3-GFP, PBI121-BpJAZ5-GFP, PBI121-BpJAZ6-GFP vectors were transformed into GV3101 [73]. We injected the pBI121-BpJAZs-GFP vectors and pBI121-GFP vector that were transferred into the *Agrobacterium* strain into tobacco leaves. The injected tobacco was cultured in dark for 24–36 h. Then, the epidermises were injected with 100 ng/mL DAPI (staining the nucleus). After 5 min, tobacco leaves were observed green fluorescence protein signal (GFP) under the confocal laser scanning microscope (LSM 800, Zeiss, Germany). The primers for gene cloning and vector construction were shown in Table S6.

We selected three BpJAZ proteins with the highest expression levels under MeJA treatment and performed yeast two-hybrid with the constructed vector. Primer sequences are shown in Table S6. Based on 6 fusion vectors, we had 9 combinations(pGBKT7-BpJAZ3/pGADT7-BpJAZ3; pGBKT7-BpJAZ3/pGADT7-BpJAZ5; pGBKT7-BpJAZ3/pGADT7-BpJAZ6; pGBKT7-BpJAZ5/pGADT7-BpJAZ5; pGBKT7-BpJAZ5/pGADT7-BpJAZ3; pGBKT7-BpJAZ5/pGADT7-BpJAZ6; pGBKT7-BpJAZ6/pGADT7-BpJAZ6; pGBKT7-BpJAZ6/pGADT7-BpJAZ3; pGBKT7-BpJAZ6/pGADT7-BpJAZ5). We used the pGBKT7-Lam/pGADT7-T vector as negative control and the pGBKT7-53/pGADT7-T vector as positive control. The six vector-constructed JAZ genes plasmids were transferred into Y2HGold yeast strain and grew on SD/-Trp /-Leu solid medium. Then, the transformed yeast cells were cultured on SD/-Trp /-Leu liquid medium and spotted in SD/-Trp /-Leu /-His /-Ade /X-α-Gal /AbA (Aureobasidin A, 125 ng/ml) on solid medium for testing interaction.

## Supplementary Information

**Additional file 1.** 

**Additional file 2.** 

**Additional file 3.**

**Additional file 4:** **Table S1.** Sequence of TIFY proteins domain

**Additional file 5:** **Table S2.** List of subfamily

**Additional file 6:** **Table S3.** List of TIFY genes from birch

**Additional file 7:** **Table S4.** Tandemly and segmentally duplicated birch TIFY gene pairs.

**Additional file 8:** **Table S5.** Syntenic gene pairs

**Additional file 9:** **Table S6.** Primer sequences ofqRT-PCR, Subcellular localization and yeast two-hybrid

**Additional file 10:** **Figure S1.** Multiple sequence alignment of PPD proteins. Multiple sequence alignment of members from birch and Arabidopsis PPD subgroup. Multiple sequence alignment was performed using BioEdit software.

**Additional file 11:** **Figure S2.** Phylogenetic analysis and multiple sequence alignment of TIFY family proteins. Phylogenetic analysis of different subfamily Arabidopsis, popular and birch proteins. Full-length amino acid sequences were use for phylogenetic analysis. The phylogenetic tree was constructed using MEGAX with the Maximum Likehood method, 1000 repetitions of bootstrap tests, and JTT matrix-based model.

**Additional file 12:** **Figure S3.** Phylogenetic analysis and multiple sequence alignment of TIFY family proteins. Phylogenetic analysis of different subfamily Arabidopsis, poplar and birch proteins. Full-length amino acid sequences were used for phylogenetic analysis. The phylogenetic tree was constructed using MrBayes 3.2.7

**Additional file 13:** **Figure S4.** Yeast two-hybrid experiments. The pGBKT7-Lam/pGADT7-T and pGBKT7-53/pGADT7-T co-transformed yeast cells were used as negative and positive control respectively.

## Data Availability

The raw sequencing data used during this study has been deposited in NCBI SRA with the accession number PRJNA856458.
