# Supplementary material for: Genome-wide identification of the TIFY family reveals JAZ subfamily function in response to hormone treatment in Betula platyphylla
Source: BMC Plant Biol. 2023 Mar 15;23:143. doi: 10.1186/s12870-023-04138-6 (PMC10015818; doi:10.1186/s12870-023-04138-6)
Supplement: Supplementary file 4 — Additional file 4: Table S1. Sequence of TIFY proteins domain [file 12870_2023_4138_MOESM4_ESM.docx]

Sequence of TIFY proteins domain

| Locus ID | TIFY domain |
| --- | --- |
| BPChr01G24987 | SASRQMTIFYGGQAHVFDDVHPNK |
| BPChr01G22907 | GTSQLTLSFRGQVYVFDSVTPEKVQAVLLLLG |
| BPChr01G22786 | SRTSELTLSFEGEVYVFPAVTPEKVQAVLLLLG |
| BPChr06G27326 | QLTLSFQGQVYVFDAVSPEKV |
| BPChr08G10696 | QMTIFYCGKVNVYDGVPLEKARGIMHLAA |
| BPChr06G11139 | APAQLTIFYAGSVSVYNDITPEKAQAIMLLAG |
| BPChr06G30991 | PERAQMTIFYAGQVIVFNEFPAEKAKEVMLLAS |
| BPChr07G30082 | TIFYNGKICVSDVTELQARTILLLAN |
| BPChr08G07947 | SPAQLTVFYAGTVHVYDDITPEKAQAIMLLAG |
| BPChr08G16644 | TAPLTIFYNGTVSVFDAPRDKAENILKLA |
| BPChr11G07023 | TAPMTIFYAGQILVFNDIPADKAREIVALAS |
| BPChr11G17878 | SSTSQLTIFYGGNVNVYNNVPQDKAQAIMLLAG |

| Locus ID | CCT domain |
| --- | --- |
| BPChr01G22907 | RAASLNRFRQKRKERCFDKKVRYSVRQEVALS |
| BPChr01G22786 | RIASLVRFREKRKERCFDKKIRYTVRKEVAQS |
| BPChr06G27326 | RLASLIRFREKRKERNFDKKIRYTVRKEVAIRAICSPFSKSYSDAWTLESYAKVEWMQRNKGQFTSSK |

| Locus ID | GATA domain |
| --- | --- |
| BPChr01G22907 | PAEATCTHCGISSKSTPMMRRGPSGPRSLCNACGLFWANR |
| BPChr01G22786 | VCQHCGVSENNTPAMRRGPAGPRTLCNACGLMWANKGTLRDLSKGGRIVSM |

| Locus ID | Jas domain |
| --- | --- |
| BPChr08G10696 | QANRRASLQRFREKRKDR |
| BPChr06G11139 | PQARQASLARFLEKRKERVMHSSPY |
| BPChr06G30991 | PIARRASLHRFLEKRKDRVTAKAPY |
| BPChr07G30082 | KRSLQCFLQKRKHRIQATSPY |
| BPChr08G07947 | PQARKASLARFLEKRKERAMSAAPY |
| BPChr08G16644 | PIARRKSLQRFLEKRKERLTSVSPY |
| BPChr11G07023 | PIVRRASLHRFLEKRKNRVAAKAPY |
| BPChr11G17878 | PMARRYSIQCFLEKRRGRLTKKSPY |

Motif Consensus

| Motif Symbol | Motif Consensus |
| --- | --- |
| 1 | RRASLQRFLEKRKER |
| 2 | QLTIFYAGQVYVFD |
| 3 | EAVCRHCGISEKSTPMMRRGPAGPRTLCNACGLMWANKGTL |
| 4 | VPPEKAQAILLLAGGG |
| 5 | CFDKKIRYTVRKEVA |
| 6 | RMQRNKGQFTSSKESNGDSNW |
| 7 | GMQWQFSKKISALPQFLSFKDVQEDKPRKIVHDPLASSGFMNISTADVC |
| 8 | QHDVHLVHRPQEVKIFSGSNQ |
| 9 | PFFDNH |
| 10 | FAQKCNLLSQFLKEKGRFGDJRLGMPC |
| 11 | WEMZMEWLFFCVFDPHADVCW |
| 12 | MERDFLGLGFKEPLKVVKEEVDNDGC |
| 13 | IQKNFNVDKQGGNHFALKVYP |
| 14 | MHAAPYNFSKKSPEC |
| 15 | WGILDHDWLENSHLI |
| 16 | CKRWSW |
| 17 | CECGNP |
| 18 | DCLIPTVTSIAGITZGW |
| 19 | DFFGMZ |
| 20 | EEVCRC |
