# Supplementary material for: Genome-wide identification of the TIFY family reveals JAZ subfamily function in response to hormone treatment in Betula platyphylla
Source: BMC Plant Biol. 2023 Mar 15;23:143. doi: 10.1186/s12870-023-04138-6 (PMC10015818; doi:10.1186/s12870-023-04138-6)
Supplement: Supplementary file 5 — Additional file 5: Table S2. List of subfamily [file 12870_2023_4138_MOESM5_ESM.docx]

List of subfamily

| Locus ID | subfamily |
| --- | --- |
| BPChr01G24987 | TIFY subfamily |
| BPChr01G22907 | ZML subfamily |
| BPChr01G22786 | ZML subfamily |
| BPChr06G27326 | ZML subfamily |
| BPChr08G10696 | PPD subfamily |
| BPChr06G11139 | JAZ subfamily |
| BPChr06G30991 | JAZ subfamily |
| BPChr07G30082 | JAZ subfamily |
| BPChr08G07947 | JAZ subfamily |
| BPChr08G16644 | JAZ subfamily |
| BPChr11G07023 | JAZ subfamily |
| BPChr11G17878 | JAZ subfamily |
