# Supplementary material for: Genome-wide identification of the TIFY family reveals JAZ subfamily function in response to hormone treatment in Betula platyphylla
Source: BMC Plant Biol. 2023 Mar 15;23:143. doi: 10.1186/s12870-023-04138-6 (PMC10015818; doi:10.1186/s12870-023-04138-6)
Supplement: Supplementary file 6 — Additional file 6: Table S3. List of TIFY genes from birch [file 12870_2023_4138_MOESM6_ESM.docx]

List of TIFY genes from birch

| Gene ID | Locus ID | Chrom | ORF | CDS |
| --- | --- | --- | --- | --- |
| BpTIFY1 | BPChr01G24987 | 1 | 482 | 1449 |
| BpZML1 | BPChr01G22907 | 1 | 385 | 1158 |
| BpZML2 | BPChr01G22786 | 1 | 519 | 1560 |
| BpZML3 | BPChr06G27326 | 6 | 542 | 1629 |
| BpPPD1 | BPChr08G10696 | 8 | 428 | 1287 |
| BpJAZ1 | BPChr06G11139 | 6 | 383 | 1152 |
| BpJAZ2 | BPChr06G30991 | 6 | 275 | 828 |
| BpJAZ3 | BPChr07G30082 | 7 | 131 | 396 |
| BpJAZ4 | BPChr08G07947 | 8 | 391 | 1176 |
| BpJAZ5 | BPChr08G16644 | 8 | 186 | 561 |
| BpJAZ6 | BPChr11G07023 | 11 | 261 | 786 |
| BpJAZ7 | BPChr11G17878 | 11 | 192 | 579 |
