# Supplementary material for: Genome-wide identification of the TIFY family reveals JAZ subfamily function in response to hormone treatment in Betula platyphylla
Source: BMC Plant Biol. 2023 Mar 15;23:143. doi: 10.1186/s12870-023-04138-6 (PMC10015818; doi:10.1186/s12870-023-04138-6)
Supplement: Supplementary file 7 — Additional file 7: Table S4. Tandemly and segmentally duplicated birch TIFY gene pairs. [file 12870_2023_4138_MOESM7_ESM.docx]

Tandemly and segmentally duplicated birch TIFY gene pairs.

| Gene ID | Gene ID | Duplication Type |
| --- | --- | --- |
| BPChr01G22907 | BPChr01G22786 | Tandem duplication |
| BPChr06G30991 | BPChr11G07023 | Segmental duplication |
