# Supplementary material for: Genome-wide identification of the TIFY family reveals JAZ subfamily function in response to hormone treatment in Betula platyphylla
Source: BMC Plant Biol. 2023 Mar 15;23:143. doi: 10.1186/s12870-023-04138-6 (PMC10015818; doi:10.1186/s12870-023-04138-6)
Supplement: Supplementary file 8 — Additional file 8: Table S5. Syntenic gene pairs [file 12870_2023_4138_MOESM8_ESM.docx]

Syntenic gene pairs

| Gene ID | Gene ID |
| --- | --- |
| BPChr01G24987 | AT4G32570.1 |
| BPChr01G24987 | Potri.006G247500.2 |
| BPChr01G24987 | Potri.018G033700.1 |
| BPChr06G11139 | Potri.012G044900.1 |
| Potri.001G062500.2 | BPChr08G16644 |
| Potri.003G165000.1 | BPChr08G16644 |
| Potri.006G139400.2 | BPChr06G30991 |
