# Supplementary material for: Genome-wide identification of the TIFY family reveals JAZ subfamily function in response to hormone treatment in Betula platyphylla
Source: BMC Plant Biol. 2023 Mar 15;23:143. doi: 10.1186/s12870-023-04138-6 (PMC10015818; doi:10.1186/s12870-023-04138-6)
Supplement: Supplementary file 9 — Additional file 9: Table S6. Primer sequences ofqRT-PCR, Subcellular localization and yeast two-hybrid [file 12870_2023_4138_MOESM9_ESM.docx]

Primer sequences of qRT-PCR, Subcellular localization and yeast two-hybrid

| Name | 5’ primers | 3’ primers |
| --- | --- | --- |
| BpJAZ 1 | ATGGAGAGAGACTTTCTGGG | CAGAATATGGTTTCTGGCTAG |
| BpJAZ 2 | ATGTCCAGCTCGCCGGAG | CATCACCCAGTTTCTCATTG |
| BpJAZ3 | ATGAGGAGGAACTGCAACTTG | CTATTAGCAAGCAGTAGAATG |
| BpJAZ 4 | ATGGAGAGAGATTTCTTGGG | CTTAGACCTATCACAAGCATC |
| BpJAZ 5 | ATGTTGAAGGTCGGTAAGGCAC | CACAGTGTTTTGTCTCAAAG |
| BpJAZ6 | ATGTCGAAGGCAACCGTTGAG | GTACAGGAGGAGTATAGACAG |
| BpJAZ7 | GACTTAGGTGTGATGCATTAC | CATTGGATGGAATACCTTCTTG |
| Bp18S | ATCTTGGGTTGGGCAGATCG | CATTACTC CGATCCCGAAGG |
| pBI121-BpJAZ3-GFP | CCGGGTCGACTGAATGAGGAGGAACTGCAACTTG | CTCACCATACTAGTATGATTGTATGGTGATG |
| pBI121-BpJAZ5-GFP | CCGGGTCGACTGAATGTCGAAGGCAACCGTTGAGV | CTCACCATACTAGT CCTCTCTTTGCGCTT |
| pBI121-BpJAZ6-GFP | CCGGGTCGACTGA ATGTTGAAGGTCGGTAAGGCAC | CTCACCATACTAGT TAACTTAAGCTCGAGCTG |
| pGBKT7-BpJAZ3 | CGAATTCCCGGGCATGAGGAGGAACTGCAACTTG | GGTCGACGGATCCTAATGATTGTATGGTGATG |
| pGBKT7-BpJAZ5 | CGAATTCCCGGGCATGTCGAAGGCAACCGTTGAG | GGTCGACGGATCTTACCTCTCTTTGCGCTT |
| pGBKT7-BpJAZ6 | CGAATTCCCGGGCATGTTGAAGGTCGGTAAGGCAC | GGTCGACGGATCTTATAACTTAAGCTCGAGCTG |
| pGADT7-BJAZ3 | CGAATTCCCGGGCATGAGGAGGAACTGCAACTTG | GGTCGACGGATCCTAATGATTGTATGGTGATG |
| pGADT7-BJAZ 5 | GATCCATCGAGCTATGTCGAAGGCAACCGTTGAG | CATCTGCAGCTCGTTACCTCTCTTTGCGCTT |
