# Supplementary material for: Genome-wide identification of the TIFY family reveals JAZ subfamily function in response to hormone treatment in Betula platyphylla
Source: BMC Plant Biol. 2023 Mar 15;23:143. doi: 10.1186/s12870-023-04138-6 (PMC10015818; doi:10.1186/s12870-023-04138-6)
Supplement: Supplementary file 10 — Additional file 10: Figure S1. Multiple sequence alignment of PPD proteins. Multiple sequence alignment of members from birch and Arabidopsis PPD subgroup. Multiple sequence alignment was performed using BioEdit software. [file 12870_2023_4138_MOESM10_ESM.pdf]

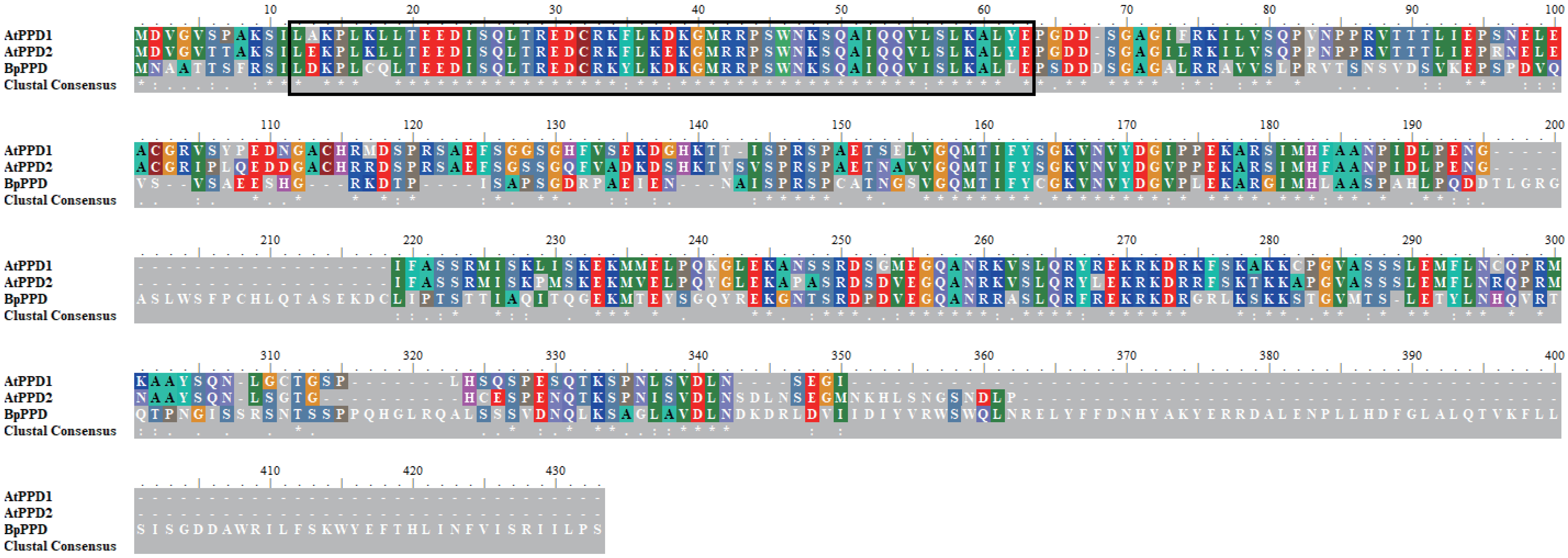

Figure S1 Multiple sequence alignment of PPD proteins. Multiple sequence alignment of members from birch and *Arabidopsis* PPD subgroup. Multiple sequence alignment was performed using BioEdit software.
