# Supplementary material for: Genome-wide identification of the TIFY family reveals JAZ subfamily function in response to hormone treatment in Betula platyphylla
Source: BMC Plant Biol. 2023 Mar 15;23:143. doi: 10.1186/s12870-023-04138-6 (PMC10015818; doi:10.1186/s12870-023-04138-6)
Supplement: Supplementary file 11 — Additional file 11: Figure S2. Phylogenetic analysis and multiple sequence alignment of TIFY family proteins. Phylogenetic analysis of different subfamily Arabidopsis, popular and birch proteins. Full-length amino acid sequences were use for phylogenetic analysis. The phylogenetic tree was constructed using MEGAX with the Maximum Likehood method, 1000 repetitions of bootstrap tests, and JTT matrix-based model. [file 12870_2023_4138_MOESM11_ESM.pdf]

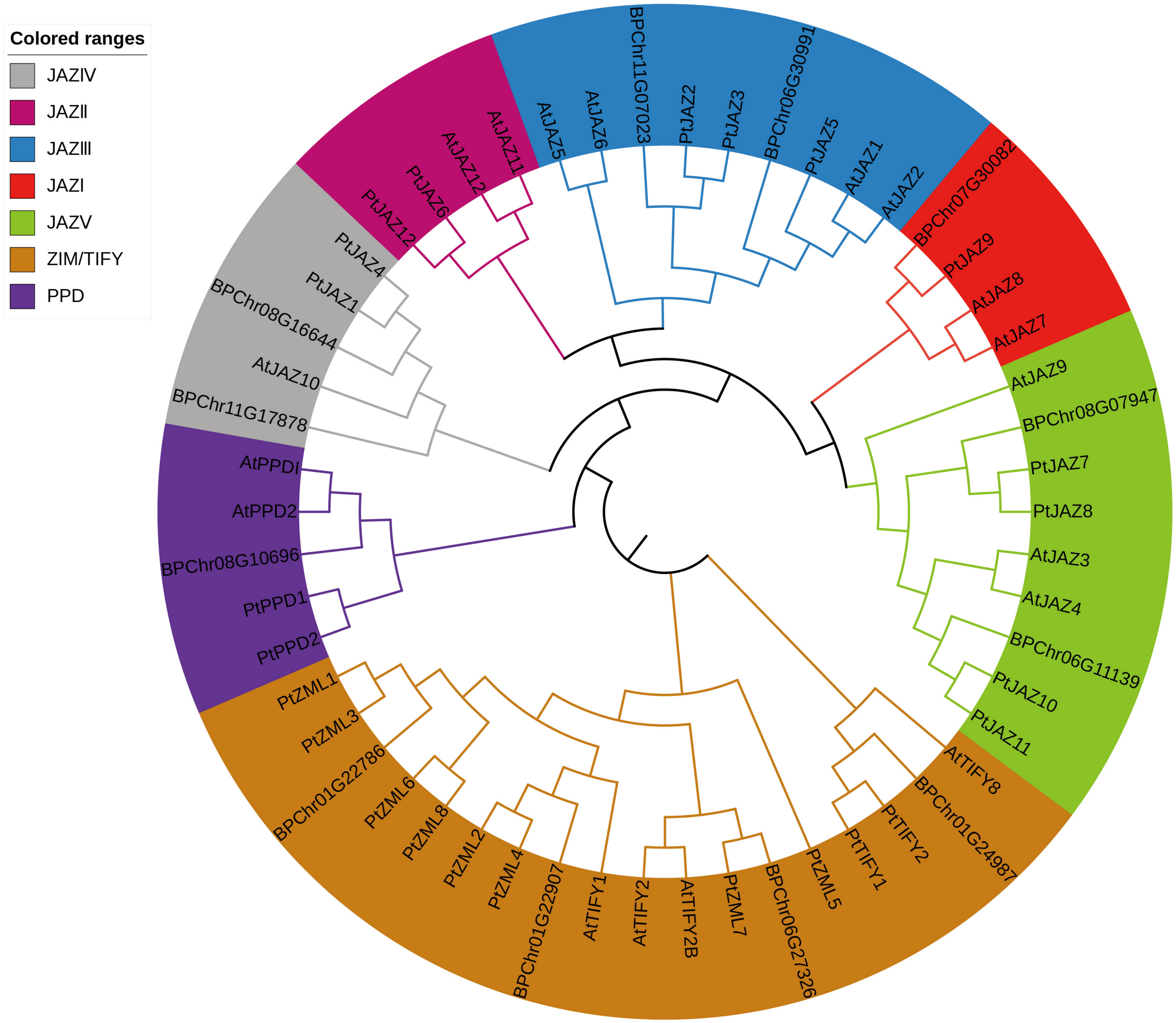

Figure S2 Phylogenetic analysis and multiple sequence alignment of TIFY family proteins. Phylogenetic analysis of different subfamily *Arabidopsis*, poplar and birch proteins. Full-length amino acid sequences were used for phylogenetic analysis. The phylogenetic tree was constructed using MEGAX with the Maximum Likelihood method, 1000 repetitions of bootstrap tests, and JTT matrix-based model.
