# Supplementary material for: Genome-wide identification of the TIFY family reveals JAZ subfamily function in response to hormone treatment in Betula platyphylla
Source: BMC Plant Biol. 2023 Mar 15;23:143. doi: 10.1186/s12870-023-04138-6 (PMC10015818; doi:10.1186/s12870-023-04138-6)
Supplement: Supplementary file 12 — Additional file 12: Figure S3. Phylogenetic analysis and multiple sequence alignment of TIFY family proteins. Phylogenetic analysis of different subfamily Arabidopsis, poplar and birch proteins. Full-length amino acid sequences were used for phylogenetic analysis. The phylogenetic tree was constructed using MrBayes 3.2.7 [file 12870_2023_4138_MOESM12_ESM.pdf]

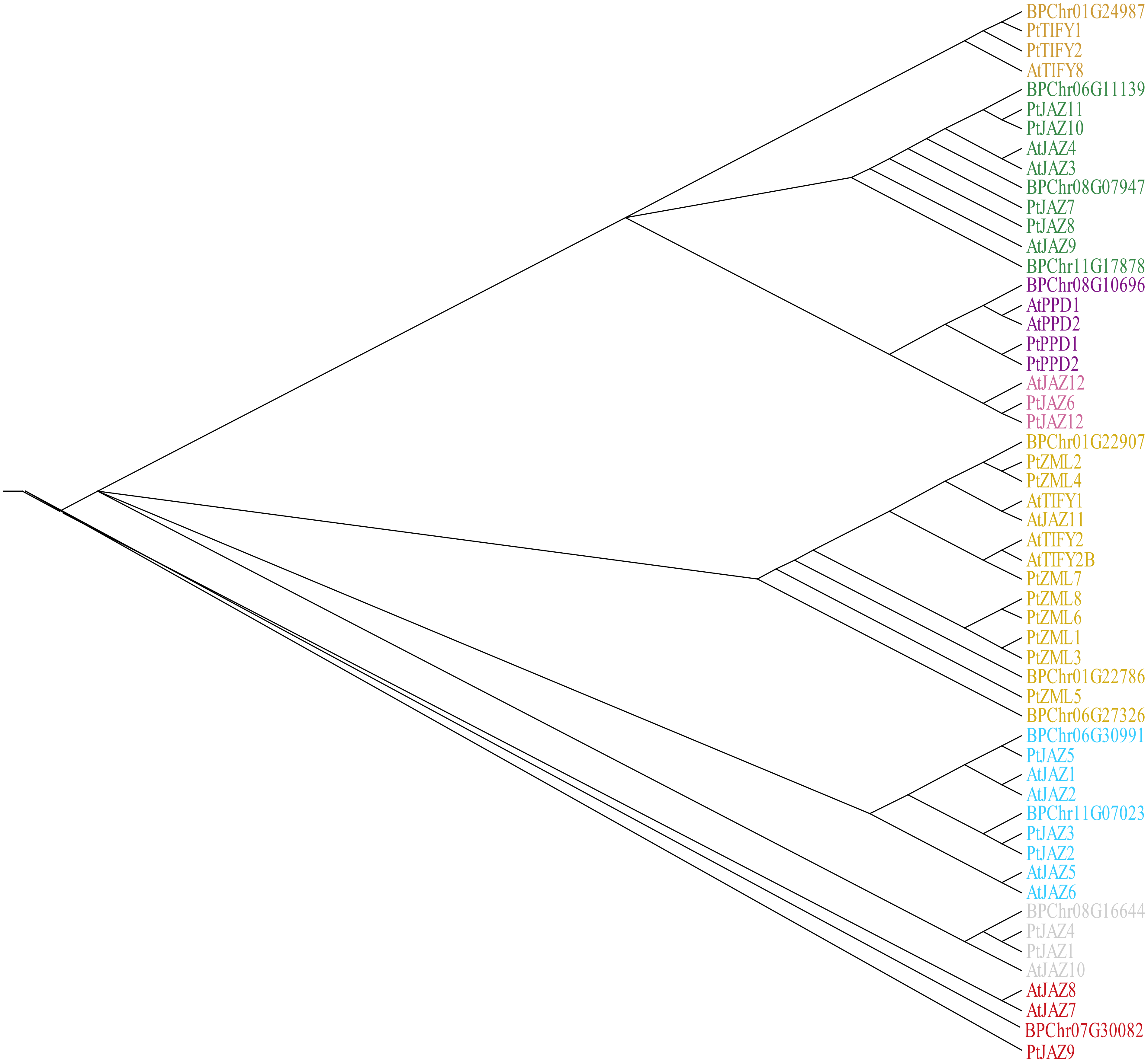

Figure S3 Phylogenetic analysis and multiple sequence alignment of TIFY family proteins. Phylogenetic analysis of different subfamily *Arabidopsis*, poplar and birch proteins. Full-length amino acid sequences were used for phylogenetic analysis. The phylogenetic tree was constructed using MrBayes 3.2.7
