# Supplementary material for: Genome-wide identification of the TIFY family reveals JAZ subfamily function in response to hormone treatment in Betula platyphylla
Source: BMC Plant Biol. 2023 Mar 15;23:143. doi: 10.1186/s12870-023-04138-6 (PMC10015818; doi:10.1186/s12870-023-04138-6)
Supplement: Supplementary file 13 — Additional file 13: Figure S4. Yeast two-hybrid experiments. The pGBKT7-Lam/pGADT7-T and pGBKT7-53/pGADT7-T co-transformed yeast cells were used as negative and positive control respectively. [file 12870_2023_4138_MOESM13_ESM.pdf]

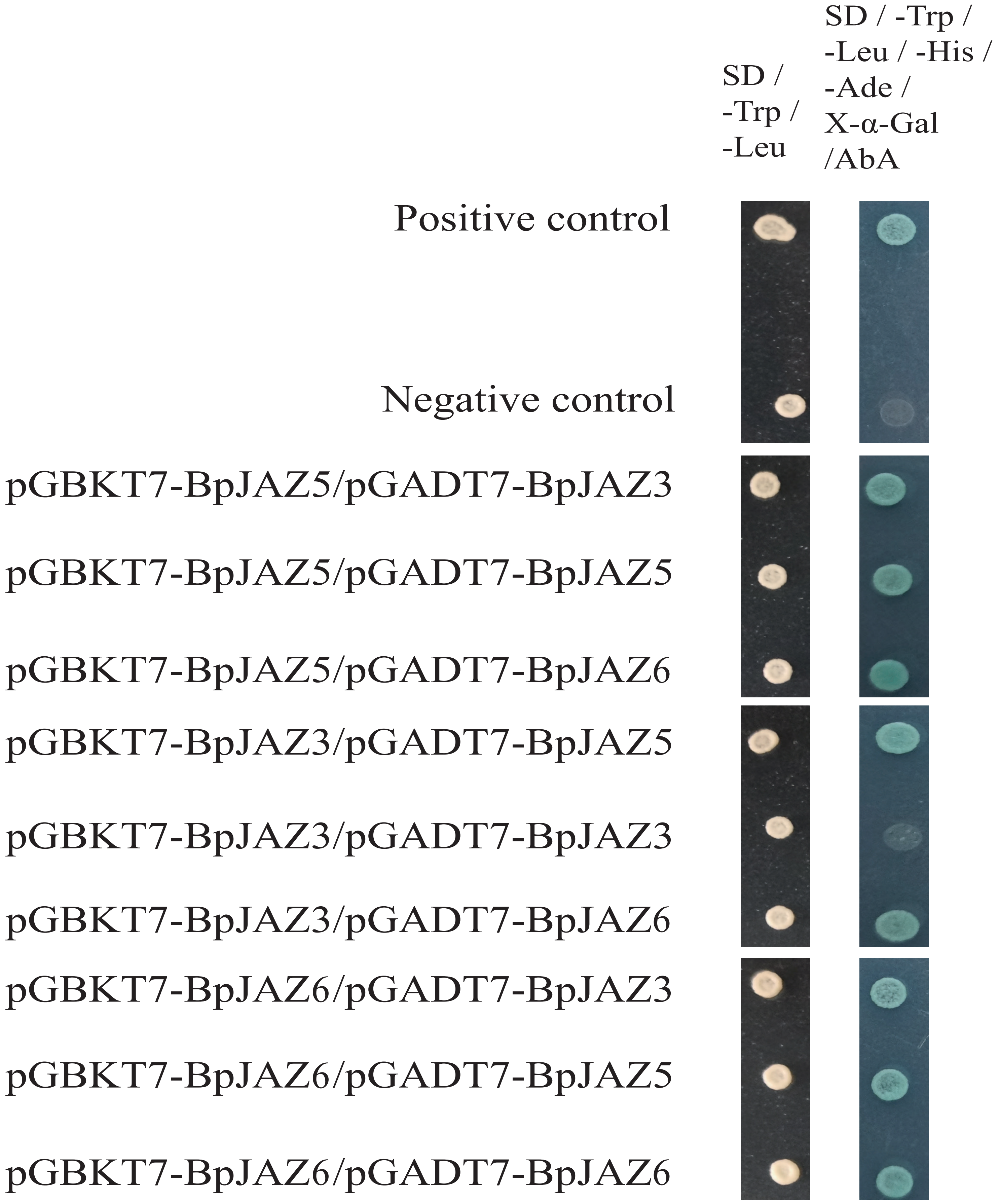

Figure S4 Yeast two-hybrid experiments. The pGBKT7-Lam/pGADT7-T and pGBKT7-53/pGADT7-T co-transformed yeast cells were used as negative and positive control respectively.
